# Impact of High-Temperature Stress on Maize Seed Setting: Cellular and Molecular Insights of Thermotolerance

**DOI:** 10.3390/ijms26031283

**Published:** 2025-02-02

**Authors:** Zhaoyi Fan, Haoqi Song, Mengyuan Qi, Mengqing Wang, Yunfeng Bai, Yuhui Sun, Haidong Yu

**Affiliations:** College of Life Sciences, Henan Agricultural University, Zhengzhou 450002, China

**Keywords:** maize, high-temperature stress, thermotolerance, seed setting, source–sink balance, signal transduction, genetic improvement

## Abstract

Global warming poses a significant threat to crop production and food security, with maize (*Zay mays* L.) particularly vulnerable to high-temperature stress (HTS). This review explores the detrimental impacts of elevated temperatures on maize development across various growth stages, analyzed within the source–sink framework, with a particular focus on seed setting and yield reduction. It provides a broad analysis of maize cellular and molecular responses to HTS, highlighting the key roles of plant hormone abscisic acid (ABA) signaling, calcium signaling, chloroplast, and the DNA damage repair (DDR) system in maize. HTS disrupts ABA signaling pathways, impairing stomatal regulation and reducing water-use efficiency, while calcium signaling orchestrates stress responses by activating heat shock proteins and other protective mechanisms. Chloroplasts, as central to photosynthesis, are particularly sensitive to HTS, often exhibiting photosystem II damage and chlorophyll degradation. Recent studies also highlight the significance of the DDR system, with genes like *ZmRAD51C* playing crucial roles in maintaining genomic stability during reproductive organ development. DNA damage under HTS conditions emerges as a key factor contributing to reduced seed set, although the precise molecular mechanisms remain to be fully elucidated. Furthermore, the review examines cutting-edge genetic improvement strategies, aimed at developing thermotolerant maize cultivars. These recent research advances underscore the need for further investigation into the molecular basis of thermotolerance and open the door for future advancements in breeding thermotolerant crops.

## 1. Introduction

The rapid increase in greenhouse gas emissions, industrialization, deforestation, and urbanization, primarily due to human activities, has led to a significant rise in global temperatures. Between 2011 and 2020, global surface temperatures averaged 1.1 °C higher than the pre-industrial period of 1850–1900, with projections indicating this increase may exceed 1.5 °C by 2040 [1]. This warming trend poses a severe threat to agricultural food security [2,3], intensifying the impact of high-temperature stress (HTS) on key staple crops such as maize, rice, and wheat [4,5,6,7]. Research suggests that every 1 °C rise in global average temperature is estimated to reduce the global yield, with maize decreasing by 7.4% and wheat by 6.0% [2], rice yields by 3.2% [2,8], and major crop yields could experience at least a loss of 3.1% [9], placing considerable pressure on global food systems. Climate models predict that HTS events will become more frequent and intense, exacerbating challenges to crop production and national food security [10,11].

A balanced source-to-sink carbon flow is essential for optimal crop yields. However, HTS disrupts this balance by impairing carbon assimilation, storage, transport, and deposition in grains, ultimately reducing yield [12,13]. HTS affects the source by decreasing the photosynthetic capacity of leaves and accelerating premature leaf senescence, which reduces the total photosynthetic area and shortens the period for carbohydrate production [14]. At the same time, developing kernels experience reduced carbohydrate metabolism due to inhibited activity of key starch biosynthesis enzymes, such as ADP-glucose pyrophosphorylase (AGPase) [15]. This results in fewer and smaller kernels, directly reducing yield [16,17]. A weakened source cannot supply enough carbohydrates, and a compromised sink cannot efficiently use what is available [17,18]. This decoupling is especially severe during the reproductive stage, where HTS simultaneously reduces photosynthesis (source) and kernel development (sink) [17]. In maize, HTS-induced reductions in photosynthesis lead to carbohydrate shortages in developing kernels, often causing kernel abortion. This process is further exacerbated by hormonal imbalances triggered by stress [19].

To withstand HTS, plants initiate a series of well-coordinated defense mechanisms that address cellular damage and restore homeostasis. The first line of defense involves maintaining membrane stability to preserve cellular integrity and scavenging reactive oxygen species (ROS) to prevent oxidative damage [20,21,22,23]. This is supported by the enhanced production of antioxidants and the accumulation of compatible solutes, which stabilize proteins and cellular structures under stress conditions. Signal transduction pathways, such as mitogen-activated protein kinase (MAPK) and calcium-dependent protein kinase (CDPK) cascades, play a central role in perceiving stress signals and activating downstream responses. Among these, the activation of heat shock transcription factors (HSFs) is critical, as they orchestrate the transcription of heat-responsive genes. This leads to the accumulation of heat shock proteins (HSPs), which act as molecular chaperones to protect and refold damaged proteins. Additionally, these responses involve the activation of antioxidant systems, and the regulation of genes related to photosynthesis and metabolism, ensuring that energy production and carbon assimilation are adjusted to meet the plant’s needs under stress [20,21,22,23]. However, recent studies have indicated conservative and differential regulations of HTS response in major crop plants such as rice, wheat and maize [24]. The thermotolerance of maize to HTS under field conditions is a multifaceted trait governed by the interplay of numerous genes, each contributing subtle but critical effects. HTS triggers a series of molecular responses in maize, including the accumulation of HSPs, activation of antioxidant systems, signal transduction, and changes in the expression of genes related to photosynthesis and metabolism [4,25].

Although extensive research has explored the molecular mechanisms underlying maize responses to HTS, many gaps remain due to the complexity of environmental conditions in the field. This review focuses on the specific threat HTS poses to global maize production, emphasizing its impact on both vegetative and reproductive growth stages critical for maize development within the source–sink framework. We provide a comprehensive assessment of the molecular mechanisms regulating maize tolerance to HTS, particularly concerning seed-set—a key determinant of yield [26]. Seed-set refers to the formation of kernels following pollination and fertilization and is crucial for overall maize productivity. Successful seed-set depends on factors such as effective pollination, fertilization, and proper development of ovules into kernels. Adverse environmental conditions, especially high temperatures during the flowering stage, can lead to incomplete seed-set, resulting in kernel abortion and substantial yield reductions [17,27]. In this review, we propose future research directions to deepen our understanding of thermotolerant mechanisms in maize, focusing on abscisic acid (ABA) and calcium signaling pathways, as well as chloroplast function and DNA damage repair (DDR). We also discuss genetic improvement strategies to develop thermotolerant maize cultivars capable of withstanding future climatic challenges.

## 2. Effects of HTS on Maize Vegetative and Reproductive Growth Within the Source–Sink Framework

**Figure 1:**
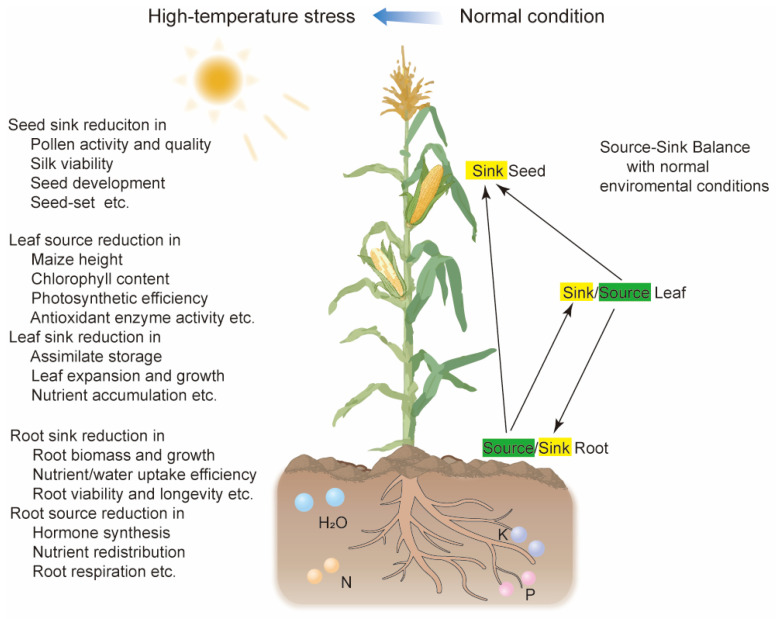
Impacts of high-temperature stress on maize seed set within the source–sink framework during reproductive and grain-filling stages. The right half of the figure illustrates that under normal temperature conditions, a dynamic and efficient source–sink balance is achieved among the source–sink root, sink–source leaf, and sink seed during the normal development of the plant, ultimately ensuring maize seed set (yield). The left half of the figure summarizes that under HTS, the seed sink strength, leaf source activity–sink strength, and root sink strength–source activity are significantly suppressed, disrupting the source–sink balance and ultimately leading to a substantial reduction in maize seed set (yield). Visualization of signaling pathways was performed using Adobe Illustrator 2023.

During the daytime, the optimal temperature for maize growth ranges from 25 to 33 °C, while at night, it ranges from 17 to 23 °C [13]. Generally, HTS occurs when ambient temperatures exceed the optimal range for plant growth by 10–15 °C [20]. This rapid increase can lead to irreversible damage during both vegetative and reproductive phases, profoundly affecting multiple physiological processes and ultimately reducing crop productivity. In maize leaves, HTS impairs photosynthesis by damaging thylakoid membranes, inhibiting Rubisco activity, and inducing photoinhibition, while excessive ROS accumulation exacerbates oxidative damage to lipids, proteins, and DNA [13,28]. HTS compromises membrane stability, resulting in ion leakage and cellular dysfunction [4]. Additionally, it disrupts hormonal balance, including an increase in ABA levels [29]. In reproductive tissues, HTS reduces pollen viability, stigmatic receptivity, and kernel development, as well as disrupting carbohydrate metabolism in developing kernels due to impaired starch biosynthesis [30,31]. In maize, HTS disrupts all growth stages, extending grain-filling time, causing grain abortion, reducing seed set, and significantly lowering overall yields [32]. The source–sink framework is fundamental to understanding plant growth and development. In maize, sources are organs that produce assimilates—primarily mature leaves through photosynthesis. Sinks are organs that consume assimilates for growth and storage, such as kernels, roots, and developing tissues like young leaves. Roots also function as the source and play a crucial role by absorbing minerals (nitrogen, phosphorus, potassium) and water, which are essential to assimilate production (Figure 1) [12,33]. HTS disrupts this delicate balance by impairing both source and sink functions during the vegetative and reproductive phases [12,33].

During the vegetative phase, HTS leads to a decrease in the leaf area index and leaf elongation rate [4,34,35]. These structural alterations, coupled with reductions in chlorophyll and antioxidant enzyme activity, diminish the photosynthetic capacity of the leaves [36,37]. As a result, the source strength of leaves is weakened, accelerating leaf senescence and leading to slower growth and wilting [38,39]. Conversely, roots have a dual role in the source–sink framework. They act as major sinks by consuming assimilates for their own growth. Simultaneously, they function as sources by absorbing minerals and water from the soil, supplying these essential resources to leaves and other sink tissues for further growth and development. HTS adversely affects root development by reducing root dry weight and inhibiting the growth of both primary roots and lateral roots [40,41]. When root zone temperatures exceed 36 °C, the water conduction capacity of the root system in the ‘thermosensitive’ maize type is significantly reduced. In contrast, the ‘thermotolerant’ maize type performs better under these conditions, likely due to its stronger root system, which ensures more efficient water absorption and transport [42,43]. This affects both the sink strength of the root to utilize assimilates and the source activity of the root to absorb minerals and water [44,45], which further indirectly impacts the source of the leaf by restricting the availability of essential nutrients required for photosynthesis [32]. The disruption of the source–sink balance under HTS meanwhile leads to an imbalance in assimilate allocation. Reduced sink demand from impaired roots can cause an accumulation of photosynthates in the leaves, potentially leading to feedback inhibition of photosynthesis [46,47]. This negative feedback may further suppress the source activity of the leaf, creating a cyclical decline in overall plant vigor. Consequently, the compromised interaction between sources and sinks under HTS might negatively impact plant growth and yield, and the plant may become less efficient at assimilating partitioning, further diminishing its ability to cope with stress (Figure 1).

Maize is particularly sensitive to HTS during the reproductive stage, especially during the microspore tetrad stage of pollen development and flowering. Even brief periods of elevated temperature can cause substantial grain loss [48,49]. During the reproductive stages, the primary source leaves experience increased respiration rates and reduced photosynthetic efficiency under HTS [14,50]. This diminishes the overall supply of carbohydrates and sugars available for reproductive development and grain-filling, weakening the source strength and limiting the energy resources needed for processes like pollen development and grain filling [25,51]. HTS accelerates heading, shortens the pollen-shedding period, and inhibits pollen tube growth [52,53], resulting in reduced pollen germination and viability [52,54]. Additionally, it disrupts anther dehiscence and delays the development of male and female reproductive structures, causing asynchronous pollination [54,55,56,57]. This misalignment leads to abnormal fertilization, kernel abortion, reduced seed-set, and ultimately decreased yields [52,53,54,55,56,57,58]. The reduced availability of assimilates from the leaves exacerbates these issues by failing to meet the heightened sink demand during reproduction (Figure 1). The advancements in research on maize yield penalties are comprehensively summarized in Table S1.

One study suggests that while nighttime HTS does not significantly impact flowering time or silk interval, it significantly increases leaf respiration and shortens the duration of pollen shedding as well as reducing pollen viability [59]. Additionally, nighttime HTS has no lasting effects on daytime leaf photosynthesis, biomass production, or assimilate transport [59]. However, another study, conducted with different maize hybrids, indicates that nighttime HTS can lead to a reduction in crop growth rate, closely associated with increased night respiration and decreased net photosynthesis at the leaf level during the day [60]. This highlights that the decline in source activity exacerbates the shortage of assimilates during critical periods of pollen development and grain filling. Consequently, high nighttime temperatures during flowering can significantly reduce seed-set in maize by disrupting the delicate balance between source supply and sink demand.

## 3. Quantitative Trait Loci Mapping (QTLs), Genome-Wide Association Studies (GWAS), and Molecular Markers for Enhancing Thermotolerance in Maize

HTS triggers complex responses in plants, involving numerous cellular components and polygenic traits at the whole-plant level. In maize, a single exposure to HTS can activate the expression of over 5000 genes [61]. Research into the molecular mechanisms of maize thermotolerance primarily focuses on two key areas. First, high-throughput omics and multi-omics approaches are employed to identify thermotolerance-related genes on a global scale [62,63,64,65,66,67]. The wealth of accumulated omics data provides a robust foundation for understanding the molecular mechanisms underlying thermotolerance. Second, due to the complexity of field conditions, genetic research uses established thermotolerance indicators to identify key QTLs [68,69] and genetic variations such as single nucleotide polymorphisms (SNPs) and insertions/deletions (InDels), often through GWAS [70,71]. These studies facilitate the cloning of relevant genes and offer deeper insights into their molecular functions, enriching our understanding of the regulatory networks involved in maize thermotolerance [27,72]. This approach is also effective for screening and breeding new thermotolerant maize cultivars.

Classical quantitative genetics approaches use pollen viability and pollen-shedding characteristics of maize tassels as indicators to study the genetic mechanisms of thermotolerance related to yield [72,73,74]. Through QTL mapping based on parental populations, several QTLs related to pollen germination and pollen tube growth under HTS have been identified [68,69]. For example, Frey et al. [75] identified 11 QTLs associated with yield thermotolerance using 6 segregating populations from Dent and Flint maize. Additionally, an association analysis using the expression level of the gene *ZmbZIP60* revealed a major-effect QTL that regulates maize thermotolerance by influencing ZmbZIP60 expression [76]. Male sterility in plants, defined as the abnormal development of male organs leading to non-functional pollen, has been extensively studied. Although over 200 male-sterile mutants have been discovered, only about a dozen nuclear male sterility genes have been cloned, and the mechanisms of most remain unclear [77,78,79]. Significant progress has been made in understanding photothermosensitive and thermosensitive male sterility mutants or QTLs in plants [80,81], laying the groundwork for identifying genetic structures that influence maize seed-set under HTS conditions.

Compared to traditional QTL mapping, GWAS offers higher resolution and can identify genes associated with specific traits within a single population [70,71]. GWAS has facilitated the development of a large number of SNPs, which are instrumental in understanding trait associations [82]. Leaf scorch during flowering is another important thermosensitive phenotype observed in maize under HTS. By using eight populations derived from a cross between the inbred line Abe 2, which exhibits no leaf scorch, and B73, which is prone to leaf scorch, researchers developed 10,112 polymorphic markers via specific locus-amplified fragment sequencing (SLAF-seq). This analysis identified six candidate genes associated with thermotolerance of leaves during flowering [83].

Combining GWAS with linkage mapping has proven to be a powerful strategy for pinpointing gene loci associated with thermotolerance in maize [84]. Two genetic studies have identified key candidate genes involved in the thermotolerance of maize during the reproductive stage using established thermotolerant indicators and integrating linkage and association analyses [27,72]. In one study, researchers employed GWAS alongside a recombinant inbred line (RIL) population derived from a thermosensitive maize inbred line BT-1 and a thermotolerant line N6 [27]. Thermotolerant seed-set was evaluated across three years, two locations, and six environments, leading to the identification of thermotolerance QTLs and candidate genes closely linked to seed-set and pollen activity traits. Using seed set as a key indicator, the study identified 17 candidate genes for thermotolerant seed set [27]. Another study performed by combination of QTL and GWAS identified 37 candidate genes related to maize anthers thermotolerance [72]. These advancements provide a foundation for breeding more thermotolerant maize cultivars and understanding the molecular mechanisms of maize thermotolerance.

Despite these advances, relatively few thermotolerance genes have been cloned and functionally characterized in maize compared to model plants like *Arabidopsis*, tomato, and rice through QTL and GWAS. Several factors contribute to the increased difficulty in maize, including its complex genome structure [85,86], rapid linkage disequilibrium (LD) decay [87], high genetic diversity [85,86], and the complexity of field conditions where numerous genotype × environment (G×E) interactions complicate the identification of key thermotolerant QTLs [88]. Moreover, the polygenic nature of thermotolerance, influenced by a multitude of environmental factors, requires large and diverse mapping populations to capture rare alleles with significant effects, complicating the identification of robust QTLs for thermotolerance [89]. The advent of next-generation sequencing (NGS) technologies has rapidly accelerated the generation of molecular markers from maize inbred lines, landraces, and wild relatives (such as teosinte) using genotyping-by-sequencing (GBS) approaches [90,91,92]. These molecular markers facilitate high-resolution mapping, and the identification of genetic variations associated with thermotolerance.

## 4. Thermotolerance Mechanisms of Maize Seed-Set in Response to HTS

**Figure 2:**
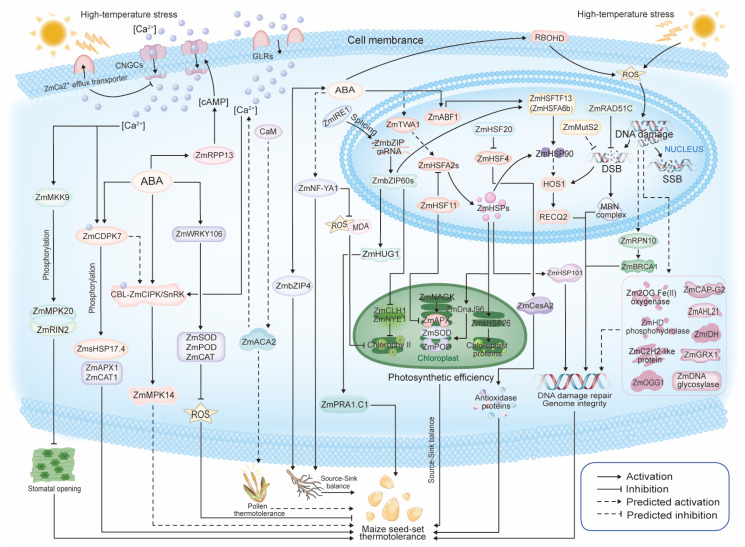
Model of molecular responses to high-temperature stress in maize.

HTS poses significant challenges to maize productivity by affecting various physiological and molecular processes crucial for seed setting. Understanding the underlying thermotolerance mechanisms is essential for developing thermotolerant maize cultivars and enhancing maize yield. Recent advancements in understanding HTS in maize have highlighted four critical targets influenced by HTS: ABA signaling, calcium signaling pathways, chloroplast function, and DDR system (Figure 2). The following sections will discuss the effects of HTS on each of these key pathways in maize. The genes critical to thermotolerance in maize are outlined in Table 1.

Based on the current research progress on the molecular mechanisms underlying thermotolerance in maize, four major signaling pathways are identified as critical mediators of seed set thermotolerance. These pathways include calcium signaling, ABA, chloroplast photosynthetic efficiency, and DDR. Calcium signaling pathway: Under HTS, membrane proteins such as cyclic nucleotide-gated channels (CNGCs) and glutamate receptor-like channels (GLRs) facilitate the influx of calcium ions into the cytoplasm, while calcium efflux transporters export calcium ions extracellularly. The elevated cytoplasmic calcium concentration activates the ZmMKK9-ZmMPK20/ZmRIN2 signaling pathway, which suppresses stomatal opening to reduce excessive water loss caused by HTS. Calcium also activates ZmCDPK7, which phosphorylates the chaperone protein ZmHSP17.4 and ROS-scavenging enzymes ZmAPX1 and ZmCAT1. Additionally, calmodulin (CaM) activates ZmACA2, which may further enhance intracellular calcium accumulation. ABA signaling pathway: ABA plays a central role in regulating maize thermotolerance and exhibits tight interconnections with calcium signaling, chloroplast photosynthetic efficiency, and DDR under HTS. High temperatures increase intracellular ABA levels, activating ZmRPP13, which elevates cytoplasmic cAMP levels and further boosts calcium influx via CNGCs. ABA directly or indirectly activates kinases such as ZmCIPK and ZmSnRK, leading to the activation of ZmMPK14. Additionally, ZmWRKY16, responsive to ABA, upregulates downstream ROS-scavenging enzymes (ZmSOD, ZmPOD, and ZmCAT) to mitigate intracellular oxidative stress caused by high temperatures. Interestingly, ABA can also promote intracellular ROS accumulation by enhancing the activity of membrane-bound RBOHD enzymes. Positive feedback exists between ABA and ZmbZIP4, amplifying the stress response. ABA may suppress ROS and malondialdehyde (MDA) accumulation via ZmNF-YA1, thereby protecting chlorophyll from degradation. Moreover, ABA likely activates the ZmTWA1-ZmHSFA2 pathway, leading to the accumulation of chloroplast-localized proteins ZmHSP26 and ZmDnaJ96, which stabilize ROS-scavenging enzymes such as ZmSOD and ZmPOD under HTS, ensuring sustained chloroplast photosynthetic efficiency. Chlorophyll degradation regulators ZmCLH1 and ZmNYE1 are negatively controlled by the nuclear transcription factor ZmbZIP60, whose protein levels are influenced by ZmIRE1 via transcriptional splicing. As a positive regulator of thermotolerance, ZmbZIP60 activates the ZmHUG1-ZmPRA1.C1 pathway. ABA signaling also activates the ZmABF1-ZmHSFTF13 (ZmHSFA6b) cascade, leading to the accumulation of ZmHSP90, which may further participate in DDR through the HOS1-RECQ2 pathway. DNA damage repair pathway: HTS causes genomic DNA damage, including DSBs and SSBs. ZmRAD51C plays roles in mitigating DSBs, and ZmMutS2 is also likely involved in this process. ZmHSFA2, through the activation of ZmHSP90, also contributes to DDR. Signals from high temperature-induced DNA damage may activate proteins such as ZmRPN10, ZmBRCA1, Zm2OG Fe(II) oxygenase, ZmCAP-G2, and ZmAHL21, among others, which may participate in DDR processes. These components form a complex regulatory network centered on ABA signaling, conferring maize with the ability to maintain kernel development under HTS. Visualization of signaling pathways was performed using Adobe Illustrator 2023.

### 4.1. Regulation of ABA Signaling Pathway Under HTS

ABA plays a central role in mediating plant stress responses by enhancing antioxidant capacity and modulating ROS levels, thereby improving thermotolerance [93]. HTS leads to excessive accumulation of ABA and suppression of cytokinin levels, disrupting proper maize kernel development [31]. On the other hand, treating maize seedlings with a calcium ion solution and ABA has been shown to enhance antioxidant enzyme activity, reduce lipid peroxidation, and improve heat tolerance [94]. ABA induces the expression of NADPH oxidase (RBOHD), leading to increased ROS production [95]. Under HTS, ABA significantly promotes maize growth [96]. Additionally, endogenous hydrogen sulfide (H_2_S) and ABA levels in maize seedlings can mutually induce each other under both normal and HTS conditions. By regulating the activities of metabolic enzymes and gene expression, the combined or individual application of H₂S and ABA significantly improves maize seedling thermotolerance, increasing survival rates and reducing membrane damage [97]. Similarly to ABA, under abiotic stress conditions, γ-Aminobutyric acid (GABA) rapidly accumulates and triggers a series of protective mechanisms that help plants cope with abiotic stress. Exogenous application of GABA has been shown to significantly improve crop performance in saline soils [98], suggesting that exploring whether GABA treatment can significantly enhance maize thermotolerance is a promising avenue for further research.

In maize, high-temperature and ABA-induced *ZmCDPK7* enhances thermotolerance by upregulating *small heat shock protein 17.4* (*sHSP17.4*), *ascorbate peroxidase 1* (*APX1*), and *catalase 1* (*CAT1*) under HTS [64]. WRKY transcription factors are vital for plant development, defense, and stress responses. Overexpression of *ZmWRKY106* enhances thermotolerance in transgenic *Arabidopsis* by regulating stress-responsive genes via the ABA signaling pathway. It also reduces ROS levels by enhancing the activities of key antioxidant enzymes such as superoxide dismutase (SOD), peroxidase (POD), and CAT, indicating its broad involvement in abiotic stress response pathways [99]. Nuclear factor Y (NF-Y) is a heterotrimeric transcription factor that is widely conserved among eukaryotes. ZmNF-YA1 is involved in maize root development. Following HTS, *ZmNF-YA1* mutants exhibit higher MDA content and ROS accumulation compared to WT and overexpression lines, along with lower chlorophyll levels. The role of ZmNF-YA1 in protein stability, refolding, and the regulation of ABA, ROS, and temperature signals may explain why its overexpression enhances thermotolerance, whereas the mutant displays a thermosensitive phenotype [100]. Additionally, basic leucine zipper (bZIP) transcription factors, like ZmbZIP4, also regulate multiple stress responses. *ZmbZIP4* is differentially expressed in various maize organs and is induced by HTS and ABA treatment. As a positive regulator of stress tolerance, ZmbZIP4 is involved in root development, and its overexpression increases ABA synthesis, enhancing maize resistance to HTS [101]. Both ZmNF-YA1 and ZmbZIP4 regulate maize thermotolerance and root development, suggesting they may function in the source–sink balance in maize seed setting under HTS. ZmRPP13-LK3, a newly identified adenylate cyclase (AC), catalyzes ATP to produce cyclic AMP (cAMP), contributing to ABA-mediated thermotolerance in maize through activating the expression of *sHSP17.2*, *sHSP17.4*, *HSP70*, and *HSP82* [102]. *ZmHSF11*, a heat shock transcription factor (*HSF*) gene in maize, belongs to the *HSF class B* and is upregulated in response to HTS. Overexpression of *ZmHSF11* in *Arabidopsis* and rice reduces the survival rate and ABA sensitivity of transgenic plants under HTS. Additionally, ZmHSF11 negatively regulates oxidative stress-related genes such as *APX2*, *DREB2A*, *HsfA2e*, and *HSP17*, leading to increased cell death under HTS [103]. Additionally, *GRMZM2G406715* encodes a bZIP transcription factor (ZmABF1) homologous to *Arabidopsis* ABA-responsive element binding factor 1 (ABF1), which activates *ZmHSFA6b*, a heat shock factor necessary for ABA-mediated thermotolerance [27,104] (Figure 2). Recent studies in *Arabidopsis* have identified Thermo-With ABA-response 1 (TWA1) as a temperature-sensing transcriptional coregulator that plays a significant role in the integration of abiotic stress responses, particularly involving ABA and HTS. The expression of *HSF A2* (*HSFA2*) and *heat shock proteins* (HSPs) (e.g., HSP90) relies on TWA1 [105] (Figure 2). By connecting the responses to HTS, TWA1 enhances the plant overall resilience to HTS, making it a vital component in the complex regulatory networks that govern thermotolerance. Homologs of *AtTWA1* in maize suggest a conserved role in temperature sensing, warranting further investigation into its function in maize thermotolerance. Further research could provide deeper insights into the molecular mechanisms underlying these interactions.

### 4.2. Calcium Signaling in Maize Response to HTS

Calcium ions (Ca^2^⁺) play a pivotal role as universal second messengers in plant cells, mediating responses to various abiotic stresses, including HTS. Calcium is a versatile second messenger that can modulate many cellular processes [106]. In maize, calcium signaling is integral to activating defense mechanisms that enhance thermotolerance (Figure 2) (Table 1). This section explores how HTS affects membrane properties, triggers calcium influx, and initiates signaling cascades leading to the expression of *HSPs* and other protective responses.

Elevated temperatures disrupt the lipid composition of the plasma membrane, increasing its fluidity and causing conformational changes in membrane-bound proteins, including calcium channels [107]. This disruption leads to the opening of Ca^2^⁺ channels, resulting in a rapid influx of Ca^2^⁺ into the cytoplasm [108]. The increase in cytosolic Ca^2^⁺ concentration acts as an initial signal of HTS. Specific calcium channels, such as cyclic nucleotide-gated ion channels (CNGCs), are activated by increased cAMP levels under HTS, facilitating further Ca^2^⁺ influx [109]. These channels amplify the calcium signaling cascade, enhancing the activation of downstream responses. Glutamate receptor-like channels (GLRs) also regulate intracellular Ca^2^⁺ concentrations by sensing external glutamate signals, thereby enhancing maize thermotolerance [110]. The elevated Ca^2^⁺ levels trigger downstream signaling pathways that activate stress-responsive genes and proteins essential for thermotolerance, such as HSFs and HSPs [109,111,112]. Calcium ions bind to specific calcium sensor proteins, such as calmodulin (CaM) and CDPKs, which transduce the signal by modifying the activity of target proteins [113]. Ca^2^⁺-activated CaM forms a complex that interacts with HSFs. This interaction enhances the DNA-binding ability of HSFs to heat shock elements (HSEs) in the promoters of *HSP* genes, upregulating their expression [113]. HSPs function as molecular chaperones, preventing protein denaturation and aggregation under HTS, thereby protecting cellular proteins and maintaining cellular homeostasis.

Gao et al. [27] and Wen et al. [83] collectively identified several candidate genes involved in calcium signaling pathways crucial for thermotolerance in maize. These discoveries, along with recent reports of other key genes (Table 1), highlight the complex network of calcium signaling in response to HTS and underscore the importance of further research in this area. *Zm00001d033334* was identified as a candidate gene associated with thermotolerance during flowering [83]. The *Arabidopsis* homolog of *Zm00001d033334* (*ZmACA2*), known as *AtACA2*, encodes a CaM-regulated Ca^2^⁺-ATPase localized in the endoplasmic reticulum (ER). Under HTS, AtACA2 plays a crucial role in maintaining calcium homeostasis and ensuring pollen transfer efficiency, which is vital for pollen thermotolerance in *Arabidopsis* [114,115]. Gao et al. [27] identified 17 candidate genes associated with thermotolerance during seed setting. Among them, the thermotolerance candidate gene *GRMZM2G023081* encodes a Ca^2+^ efflux transporter (Zm Ca^2+^ efflux transporter). *GRMZM2G409658* (*ZmCIPK*/*SnRK*) encodes a calcineurin B-like protein-interacting kinase (CIPK), also known as a sucrose non-fermentation-related protein kinase (SnRK). Members of the ZmCIPK/SnRK family are key players in pollen tube growth, seed set, and abiotic stress responses by mediating Ca^2^⁺ signaling [116,117]. The mitogen-activated protein kinase (MAPK) cascade is another critical pathway activated by calcium signaling under HTS. In maize, the MAPK cascade involving ZmMKK9-ZmMPK20-ZmRIN2 negatively regulates HTS-induced stomatal opening, balancing water loss and leaf temperature, thereby contributing to enhanced thermotolerance [118]. Another candidate gene identified by Gao et al. [27], *GRMZM2G062914*, encodes the maize MAPK 14 (ZmMPK14). Its *Arabidopsis* homolog, ABA-induced *AtMPK1*, increases sensitivity to ABA and enhances ABA-mediated stress responses [119]; and it is activated by AtSnRK2 [120]. Interaction network analysis suggests a strong connection between *ZmMPK14* and *ZmCIPK/SnRK* (*GRMZM2G409658*), indicating that Ca^2^⁺ and ABA signaling may mediate their interaction. This signaling cascade likely activates downstream genes such as *ZmABF1* and *ZmHSFA6b*, thereby enhancing maize thermotolerance [27]. Since ZmCDPK7, ZmCIPK/SnRK, and ZmMPK14 are calcium-dependent, ABA-responsive, and HTS-induced [61,117,119,121], they likely function together as a signaling module (ZmCDPK7-ZmCIPK/SnRK-ZmMPK14) (Figure 2). This module integrates calcium and ABA pathways with HTS responses in maize, coordinating a multifaced defense mechanism to enhance maize resilience under HTS. Their roles highlight significant potential as targets for improving thermotolerance in maize. Modifying their expression or activity could strengthen maize response to HTS, positioning them as promising candidates for genetic manipulation in thermotolerance breeding programs.

### 4.3. Role of Chloroplasts in Maize Thermotolerance

Chloroplasts play a pivotal role in plant thermotolerance, orchestrating a series of structural and molecular responses to HTS, which adversely affects photosynthesis and disrupts the cellular energy balance. Moderate HTS (35–40 °C) causes significant structural changes in chloroplasts, such as enlargement and increased vesicle formation, indicating alterations in thylakoid structures [122]. These changes impair the chloroplast redox state, affecting photosynthetic reactions and carbon metabolism [123].

HTS activates the RNA splicing factor inositol-requiring enzyme 1 (IRE1) located in the ER. Activated ZmIRE1 facilitates the splicing of ZmbZIP60 mRNA, leading to the production of the active ZmbZIP60 protein in maize [124]. ZmbZIP60 functions as a transcription factor that downregulates genes *CLH1* and *NYE1*, which are involved in chlorophyll degradation, thereby maintaining chlorophyll levels and preserving photosynthetic efficiency under HTS [125]. Moreover, ZmbZIP60 activates the HTS-induced molecular chaperone heat up-regulated gene 1 (*ZmHUG1*), which prevents the aggregation of preacylated RAB receptor 1.C1 (ZmPRA1.C1) and stabilizes other target proteins, alleviating ER stress and enhancing maize thermotolerance [126]. This regulatory pathway is essential for sustaining chloroplast function and preventing the detrimental effects of HTS-induced chlorophyll loss. In response to HTS, sHSPs are imported into chloroplasts to safeguard the photosynthetic apparatus (Figure 2). These molecular chaperones prevent protein denaturation and aggregation, thereby protecting the thylakoid membrane and photosystem II (PSII) from HTS-induced damage [127,128]. For example, HTS-induced ZmsHSP26 is also triggered by H₂O₂ treatment and interacts with specific chloroplast proteins, protecting them against oxidative and thermal stress [129]. Overexpression of ZmDnaJ96, a member of the DnaJ (Hsp40) family of proteins in maize, is reported to localize primarily in the chloroplasts, enhancing the activity of chloroplast antioxidant enzymes and protecting chloroplasts from HTS; while silencing ZmDnaJ96 reduces thermotolerance in maize by lowering antioxidant enzyme activity of SOD and POD [130] (Figure 2). By stabilizing key components of the photosynthetic machinery, these sHSPs ensure the continued functionality of PSII and overall photosynthetic performance under adverse temperature conditions. *ZmNAGK*, another key gene, is significantly upregulated in maize under HTS and is localized in chloroplasts. Overexpression of ZmNAGK enhances thermotolerance in tobacco during seed germination and seedling growth. Transcriptome analysis reveals that ZmNAGK regulates the expression of genes encoding antioxidant enzymes, such as *APX2* and *SODC*. and heat shock network genes [131]. Future research should focus on elucidating the intricate signaling networks between chloroplasts and other cellular components to further understand the mechanisms underlying thermotolerance.

### 4.4. DNA Damage Repair Mechanisms Under HTS

HTS leads to the overproduction of ROS in plants, resulting in oxidative stress and various forms of DNA damage, including single-strand breaks (SSBs) and double-strand breaks (DSBs) [132,133,134,135]. Excessive ROS not only causes direct damage to DNA but also impairs the function of DNA damage repair (DDR) enzymes, further compromising genome integrity [134]. This accumulation of DNA damage can disrupt cellular processes and affect plant growth and reproduction. To counteract these effects, plants activate DDR systems such as homologous recombination (HR) and non-homologous end joining (NHEJ) to repair DSBs [136]. The MRE11-RAD50-NBS1 (MRN) complex is essential for recognizing DNA damage and initiating the repair process by signaling and recruiting other repair proteins [137]. However, HTS can inhibit these repair pathways by affecting the expression and activity of key repair proteins directly or indirectly, making the maintenance of genome integrity critical for thermotolerance.

Molecular chaperones from the HSP family, particularly HSP101, play vital roles in preventing protein misfolding and supporting DNA repair signaling pathways under stress conditions [138]. In maize, overexpression of *ZmHSP101* in anthers enhances thermotolerance during microspore formation by facilitating DSB repair and ensuring proper progression of meiosis [139]. *ZmCAP-G2* in maize encodes a protein homologous to the condensing complex subunit CAP-G2, and its *Arabidopsis* homolog CAP-G2 functions in DDR [140]. Another key player in DDR is the radiation-sensitive recombinase ZmRAD51C, which is essential for meiotic DSB repair and HR in maize, ensuring the proper segregation of homologous chromosomes [141] (Figure 2).

Among the 17 candidate genes related to maize seed-set thermotolerance [27], four candidates or their homologues have been identified that contribute to DDR or genome stability under HTS. For instance, *GRMZM2G060349* (*ZmMutS2*) encodes a protein belonging to Mutator S (MutS) protein family. MutS family proteins are a part of the mismatch repair (MMR) system that recognizes and binds to mismatched nucleotides during DNA replication. Plant MutS2, unlike its well-studied MSH2 counterpart, has not been characterized extensively in terms of its molecular function in plants [142]. Studies suggest that it might play a role in DDR and protection against oxidative stress, similar to what has been observed in other organisms [142]. *ZmMutS2* is upregulated in thermotolerant maize lines in response to high temperature, indicating its protective role during reproduction under HTS [27]. *GRMZM2G341723* encodes a HD domain-containing metal-dependent phosphohydrolase (ZmHD phosphohydrolase) and is a large and diverse family of enzymes characterized by the presence of an HD (histidine–aspartate) domain, which coordinates metal ions to hydrolyze phosphodiester bonds. It has been reported that the human SAMHD1 and its probable *Arabidopsis* ortholog of VEN4 function in DSB repair by HR, which indicates functional conservation in DNA repair by VEN4 and SAMHD1 [143]. *GRMZM2G136494* encodes a C2H2-like zinc finger protein (ZmC2H2-like protein), which typically consist of a conserved sequence of two cysteines and two histidines that coordinate a zinc ion. The structural flexibility of C2H2 zinc finger domains allows them to bind to DNA, RNA, and proteins, which makes them versatile in regulating gene expression, participating in the DDR pathways and maintaining genome integrity under conditions of DNA damage caused by various stressors [144]. Human FLYWCH1 can colocalize with DNA damage markers like γH2AX and regulate the expression of key DDR proteins such as ATM and p53. This suggests that C2H2 proteins like FLYWCH1 might facilitate the recruitment and activation of DDR machinery, contributing directly to the maintenance of genome stability by promoting efficient DDR [145]. C2H2 zinc finger proteins often contain multiple functional domains, such as KRAB (Krüppel-associated box) and SCAN domains, which contribute to transcriptional repression and protein–protein interactions. These additional domains enable C2H2 proteins to regulate various aspects of cell biology, including the suppression of transposable elements, which is crucial for preserving genomic stability in *Arabidopsis* [146] (Figure 2).

Similarly, Feng et al. [72] identified 37 candidate genes related to maize anther thermotolerance. Among them, four candidates or their homologs have been identified that contribute to DDR or genome stability under HTS. *Zm00001d033327* encodes a 2OGFe(II)-dependent oxygenase (Zm2OGFe(II) oxygenase), which shares homology with a conserved human enzyme that catalyzes oxidative reactions and responds to cellular stresses like hypoxia and DNA damage, promoting DDR and maintaining genome integrity [147]. *Zm00001d003124* encodes a DNA glycosylase (ZmDNA glycosylase), which plays a key role in base excision repair by recognizing and excising damaged bases, initiating a repair process that restores DNA structure with high accuracy [148]. *Zm00001d028396* (*ZmRPN10*) encodes the 26S proteasome non-ATPase regulatory subunit 4-like protein. Its *Arabidopsis* homolog, *RPN10* (*AT4G38630*), is known to be induced by HTS [149]. As part of the 26S proteasome, human RPN10 helps mediate the degradation of ubiquitinated BRCA1- an essential DDR protein, involved in DDR. By regulating the stability of these proteins, RPN10 indirectly influences the efficiency and fidelity of DDR mechanisms [150]. Therefore, it might be worthwhile to examine the response of maize ZmRPN10 mutants to DNA-damaging treatments (e.g., γ-irradiation, UV light, or chemicals like methyl methanesulfonate) and to look at changes in the expression and stability of known DNA repair proteins. *Zm00001d002495* encodes an AT-hook motif nuclear-localized protein 21 (ZmAHL21). AHL proteins contain the AT-hook motif, which is known to bind to AT-rich regions of DNA, influencing chromatin structure and gene expression. The AT-hook motif is conserved across various species and is also found in other DNA-binding proteins like the high mobility group (HMG) proteins, which are associated with chromatin remodeling and DDR [151,152]. *Zm00001d003081* encodes glutaredoxin homolog1 (ZmGRX1) protein. Studies in yeast and mammals have shown that glutaredoxins are involved in DDR processes by maintaining the activity of DNA repair enzymes such as OGG1 (8-oxoguanine DNA glycosylase). They also influence the activity of other repair proteins by maintaining their redox states [153,154]. *Zm00001d003083* encodes isocitrate dehydrogenase (ZmIDH) involved in the DDR pathway, primarily through its role in cellular metabolism and redox homeostasis. In humans, IDH1 and IDH2 mutations are frequently observed in cancers such as gliomas and acute myeloid leukemia (AML). These mutations are associated with the production of 2-HG, which competitively inhibits α-KG-dependent enzymes and disrupts DDR processes [155]. In plants, IDH plays a similar role in maintaining redox balance and metabolic regulation. While direct evidence of IDH’s involvement in DDR in plants is limited, its function in producing NADPH and regulating cellular redox states [156] suggests it could have an indirect impact on the plant DDR. These findings indicate that DDR and genome stability during the reproductive stages are the main components targeted by HTS (Table 1) (Figure 2).

In *Arabidopsis*, the HSP90-HOS1-RECQ2 module is activated under HTS, promoting DNA repair and maintaining genome integrity [157]. HSP90 interacts with the E3 ubiquitin ligase HOS1 and the helicase RECQ2 to facilitate the repair of DNA damage caused by HTS. A similar mechanism may exist in maize (Figure 2), where ZmHSFA2, ZmHSFA6b, and ZmHSFTF13 (a member of ZmHSFA6b family) respond to upstream signals, such as ABA or high-temperature, and activate the expression of *ZmHSP90* [27,104,125]. Thus, specific members of the ZmHSFA2 and ZmHSFA6b families likely integrate these signals to activate the HSP90-HOS1-RECQ2 module, promoting genome stability under HTS conditions (Figure 2). This regulatory relationship makes ZmHSFA2 and ZmHSFA6b family genes promising targets for genetic manipulation to enhance thermotolerance in maize. By overexpressing or modifying *ZmHSFA2s* and *ZmHSFA6bs* to efficiently regulate the HSP90-HOS1-RECQ2 module, breeders could improve thermotolerance, particularly during the sensitive stages of seed development. Further research into these regulatory networks is essential to deepen our understanding of how these pathways support genome stability and resilience to heat stress in maize.

By reinforcing DDR mechanisms and ensuring genome integrity, plants can improve their thermotolerance, which is vital for sustaining growth and reproduction under HTS. Advances in this area could lead to the development of maize cultivars with enhanced thermotolerance to HTS, addressing the challenges posed by global climate change.

### 4.5. The Structure of Maize Cell Wall and Thermotolerance

A recent study has shown that *ZmHSF4* and *cellulose synthase A2* (*ZmCesA2*) contribute to the HTS response by promoting thermotolerance. ZmHSF4 positively regulates the expression of *ZmCesA2*, leading to increased cellulose synthesis and enhanced cell wall integrity, which helps maize seedlings withstand HTS. Conversely, ZmHSF20 acts as a negative regulator by inhibiting cellulose accumulation. It binds to the promoters of *ZmCesA2* and three class A HSFs, including ZmHSF4, suppressing their transcription. This suppression may reduce the thermotolerance of maize seedlings by weakening cell wall structure and making them more susceptible to heat damage [158]. The finding suggests that changes in cell wall structure, particularly in cellulose content, may explain the observed effects on thermotolerance in maize. The cell wall rigidity and composition could influence the maize ability to maintain cellular integrity under HTS. However, the exact connection between cell wall remodeling and the HTS response remains to be fully elucidated. Further research is needed to investigate how cell wall modifications interact with HTS signaling pathways to confer thermotolerance.

**Table 1:** Key genes implicated in the regulation of high-temperature stress responses in maize.

Number	Gene ID	Functions	References
**ABA signaling**			
1	*GRMZM2G013391* (*ZmWRKY106*)	Acted as a positive factor under drought and high-temperature stress	[99]
2	*Zm00001d018178* (*ZmbZIP4*)	Involved in root development, and its overexpression increases ABA synthesis, enhancing maize resistance to HTS	[101]
3	*Zm00001d045512* (*ZmRPP13-LK3*)	Catalyzes ATP to produce cAMP, contributing to ABA-mediated thermotolerance in maize	[102]
4	*Zm00001d027874* (*ZmNF-YA1*)	A positive regulator of drought stress response is involved in maize root development	[100]
5	*Zm00001d034433* (*ZmHSF11*)	Heat stress response	[103]
**Calcium signaling**			
6	*Zm00001d033334* (*ZmACA2*)	Involved in maintaining calcium homeostasis and pollen transfer efficiency in *Arabidopsis*	[83,114,115]
7	*Zm00001d006621* (*ZmCDPK7*)	Induced by ABA to participate in heat resistance of maize by mediating phosphorylation of sHSP17.4	[64]
8	*GRMZM2G409658* (*ZmCIPK*/*SnRK*)	The CIPK/SnRK family play a key role in pollen tube growth, fruiting, and abiotic stress by sensing and mediating Ca^2+^ signaling	[27,116,117]
9	*Zm00001d028273* (*ZmMKK9*)	Phosphorylated ZmMPK20 and enhanced the inhibitory effect of ZmMPK20 on ZmRIN2 degradation	[118]
10	*Zm00001d039141* (*ZmMPK20*)	Prevented ZmRIN2 degradation by inhibiting ZmRIN2 self-ubiquitination	[118]
11	*Zm00001d006373* (*ZmRIN2*)	Balance water loss and leaf temperature, thus enhancing plant thermotolerance	[118]
12	*GRMZM2G062914* (*ZmMPK14*)	Its *Arabidopsis* homolog AtMPK1 is induced by ABA and abiotic stresses	[27,121]
**Chloroplast**			
13	*Zm00001d046718* (*ZmbZIP60*)	Links the unfolded protein response to the heat stress response in maize	[124,125]
14	*Zm00001d045336* (*ZmHUG1*)	Relieve endoplasmic reticulum stress at high temperature	[126]
15	*Zm00001d039455* (*ZmPRA1.C1*)	MAIZE PRENYLATED RAB ACCEPTOR 1. C1 was identified as a client of ZmHUG1	[126]
16	*Zm00001d028408* (*ZmsHSP26*)	Heat stress response	[129]
17	*Zm00001d024635* (*ZmDnaJ96*)	Induced by drought, high temperature, and salt stress and regulated by abscisic acid	[130]
18	*Zm00001d002734* (*ZmNAGK*)	Modulate the expression of antioxidant-enzyme encoding genes	[131]
**DDR**			
19	*Zm00001d038806* (*ZmHSP101*)	Heat stress response HSP101 mediates thermotolerance during microsporogenesis	[139]
20	*Zm00001d033333* (*ZmCAP-G2*)	Play a role in DNA damage repair or in protecting the genome from certain genotoxic stressors	[53,140]
21	*Zm00001d033327* (*Zm2OGFe* (*II*)*-dependent oxygenase*)	Response to cellular stresses including hypoxia and DNA damage	[53,147]
22	*Zm00001d044278* (*ZmRAD51C*)	Involved in both meiotic DSB repair and homologous recombination in maize	[141]
23	*GRMZM2G060349* (*ZmMutS2*)	Involved in the DNA mismatch repair process	[27]
24	*GRMZM2G341723* (*ZmHD phosphohydrolase*)	HD domain-containing metal-dependent phosphohydrolase	[27]
25	*GRMZM2G023081* (*ZmCa^2+^ efflux transporter*)	Ca^2+^ efflux transporter	[27]
26	*GRMZM2G136494* (*ZmC2H2-like protein*)	C2H2-like zinc finger protein	[27]
27	*Zm00001d003124 ZmDNA glycosylase*	DNA glycosylase	[148]
28	*Zm00001d002495* (*ZmAHL21*)	AT-hook motif nuclear-localized protein 21 (AHL21)	[151,152]
29	*Zm00001d003081* (*ZmGRX1*)	Glutaredoxin homolog1 (GRX1) protein	[153,154]
30	*Zm00001d003083* (*ZmIDH*)	Isocitrate dehydrogenase (IDH)	[155]
**Structure Cell Wall**			
31	*Zm00001d037636* (*ZmCesA2*)	Related to heat resistance of maize seedlings	[158]
32	*Zm00001d018941* (*ZmHSF4*)	Heat stress response	[158]
33	*Zm00001d026094* (*ZmHSF20*)	Heat stress response	[158]

## 5. Genetic Improvement of Thermotolerance in Maize

Genetic engineering has become a foundational practice for developing crop cultivars with improved traits, including thermotolerance. This enhancement can be achieved through the application of exogenous compounds and the manipulation of specific genes associated with HTS responses. Exogenous application of certain compounds has been shown to enhance thermotolerance in plants by activating antioxidant systems and regulating cellular redox homeostasis. In maize, exogenous application of compounds like glutamic acid has been reported to improve seedling survival rates under HTS by reducing membrane damage and enhancing osmotic regulation [159]. Additionally, the interaction between endogenous H₂S and ABA in maize seedlings can induce improved thermotolerance by increasing survival rates and reducing membrane damage through the regulation of metabolic enzyme activities and gene expression [97]. Application of appropriate concentrations of ammonia has also been shown to increase seedling vigor and improve survival rates under HTS, indicating its potential role in promoting thermotolerance [160].

In the genetic improvement of maize for thermotolerance, both traditional and modern breeding strategies have been effectively employed to enhance resilience to HTS. A recent review provided an in-depth discussion on the applications of crossbreeding, marker-assisted selection (MAS), targeted induced local lesions in genomes (TILLING), and CRISPR-Cas9 technology in maize breeding, highlighting their potential to address the challenges posed by HTS [13]. Given the complexity of field environments, the polygenic and minor-effect nature of thermotolerance traits in maize, and the network regulation of multiple signaling pathways (such as ABA, calcium ions, and DDR), using a multigene co-transformation approach may yield transgenic maize plants with optimal thermotolerance.

Advancements in computational tools and genomics have revolutionized plant breeding, making it more efficient and cost-effective. Computer simulations and modeling play a crucial role in optimizing breeding strategies for thermotolerance. Genomic selection (GS) is a modern breeding method that uses genome-wide markers to predict the performance of untested genotypes, accelerating the breeding process [161]. By integrating GS with speed breeding (SB) techniques, genetic gains in crops can be significantly increased. Computational models simulate the breeding process, allowing researchers to predict and optimize the development of thermotolerant maize cultivars without the need for extensive field trials. Environmental economics approaches can predict unobserved genotype performance by analyzing environmental attributes at the omics scale. This method combines G×E studies with geographic information system (GIS) technology, providing data-rich insights to optimize decision-making in breeding programs [162]. Such simulations reduce the reliance on costly field trials, enhancing resource efficiency and accelerating the development of thermotolerant varieties. Deep learning frameworks, such as convolutional neural networks (CNNs), have been applied to predict plant phenotypes based on genotype information. These models can predict quantitative traits from SNP data and generate significance maps to explore genotype contributions to specific traits [163]. By simulating genetic processes and assessing various breeding strategies, computational tools help identify the most promising thermotolerant maize lines for further experimental validation.

Genetic engineering of maize faces significant challenges due to public perception and regulatory complexities. Public concerns about food safety, environmental impact, and corporate control often hinder acceptance, even though scientific evidence supports the safety and benefits of genetically engineered (GE) crops [164,165]. Fears of unintended health effects, harm to biodiversity, and the dominance of large corporations in seed production fuel skepticism [166]. Additionally, misinformation and limited scientific literacy exacerbate resistance to GE maize [167]. Addressing these concerns requires transparent science communication, stakeholder engagement, and public–private partnerships to ensure equitable access to GE technologies, particularly for resource-poor farmers [168,169]. Regulatory systems further complicate the adoption of GE maize, with stringent and inconsistent approval processes increasing costs and delaying innovation [164,170]. For example, differences in global standards can disrupt trade, as maize approved in one region may be rejected in another. The advent of genome editing technologies like CRISPR-Cas9 introduces new ethical questions, such as the unintended spread of engineered traits and ensuring fair access to these tools [171,172]. Harmonizing international regulations, integrating socio-economic assessments, and adopting gene containment strategies can help address these challenges [173,174]. By fostering collaboration among scientists, policymakers, and the public, it is possible to leverage genetic engineering to improve food security while mitigating ethical and regulatory concerns.

## 6. Conclusions and Future Outlook

The increasing frequency of extreme high-temperature events due to global warming poses a significant threat to the production and yield stability of major food crops like maize. This review highlights that maize responds to HTS through mechanisms involving source–sink balance, ABA and calcium signaling, chloroplast function, and DDR system. Despite advances in understanding the HSF-HSP signaling pathway and associated biochemical mechanisms, the impact of high temperatures on DNA stability and genome integrity in maize remains underexplored. Studies in model organisms like *Arabidopsis* have demonstrated that MRN complex and modules like HSP90-HOS1-RECQ2 play crucial roles in repairing DNA damage under HTS, particularly addressing DSBs and SSBs. Investigating whether similar molecular mechanisms operate in maize is essential for enhancing thermotolerance by safeguarding genome integrity. Recent identification of key candidate genes through QTL and GWAS strongly suggests that genomic DNA stability is a key target of HTS in maize during the reproductive stage. A deeper understanding of these DNA repair pathways could lead to the development of more thermotolerant maize cultivars.

Although significant progress has been made in understanding the molecular mechanisms underlying maize responses to HTS, knowledge gaps persist, limiting the development of thermotolerant maize varieties. Addressing these gaps requires targeted research and innovative experimental strategies, including the following: (1) Functional validation of candidate genes—Advanced genome-editing techniques, such as CRISPR-Cas9 and RNA interference (RNAi), should be utilized to validate the roles of specific thermotolerance-associated genes and their regulatory pathways. (2) High-throughput phenotyping—The development of advanced phenotyping platforms is essential for accurately quantifying HTS responses across diverse maize genotypes, enabling the identification of resilient traits under field conditions. (3) Molecular mechanisms—Further investigation into the role of DNA damage repair pathways, as well as hormonal signaling mechanisms (e.g., abscisic acid and calcium signaling), is crucial for understanding the cellular processes that confer maize thermotolerance. (4) Integrative approaches—Multi-omics strategies, including transcriptomics, proteomics, and metabolomics, should be employed to unravel the complex regulatory networks involved in thermotolerance, providing insights into novel targets for crop improvement. By addressing these key areas, future research may bridge existing gaps and accelerate the development of maize varieties with enhanced resilience to HTS.

## 7. Materials and Methods

A systematic literature review was conducted to analyze studies on maize (*Zea mays* L.) reproductive responses to HTS with a focus on seed-set and thermotolerance mechanisms. Databases including PubMed, Web of Science, Google scholar and Scopus were searched using the keywords “crop”, “maize”, “corn”, “high-temperature stress”, “heat stress”, “thermotolerance”, “seed set”, “yield”, “source-sink balance”, “QTL mapping”, “GWAS”, “vegetative stage”, “reproductive stage”, “leaf”, “chloroplast”, “photosynthesis”, “gamete”, “pollen”, “silk”, “root”, “ABA”, “calcium signaling”, “reactive oxygen species (ROS)”, “DNA damage repair (DDR) system”, “genetic improvement”, “HSF”, and “HSP”, etc.

Inclusion criteria included peer-reviewed studies published in English that reported on HTS effects during maize vegetative and reproductive stages, molecular markers associated with thermotolerance, and mechanisms such as ABA signaling, DDR pathways, or calcium signaling.

Visualization of signaling pathways was performed using Adobe Illustrator 2023.

## Supplementary Materials

The following supporting information can be downloaded at: https://www.mdpi.com/article/10.3390/ijms26031283/s1.
