# Supplementary material for: Impact of High-Temperature Stress on Maize Seed Setting: Cellular and Molecular Insights of Thermotolerance
Source: Int J Mol Sci. 2025 Feb 2;26(3):1283. doi: 10.3390/ijms26031283 (PMC11818821; doi:10.3390/ijms26031283)
Supplement: Supplementary file 1 [file ijms-26-01283-s001.zip › ijms-3367276-supplementary.pdf]

Table S1. Statistical data of maize seed setting at the cellular level under HTS.

Number	Percentage	Metric	Cultivars	Description	References
**Cellular Aspect: Pollen viability**					
1	41.7%	Fresh weight of pollen	Xianyu335		[54]
	54.5%	Fresh weight of pollen	Nongda372		[54]
2	62%	Pollen viability	ZD958 (2021)		[55]
	80%	Pollen viability	ZD958 (2022)		[55]
	50%	Pollen viability	XY335 (2021)		[55]
	80%	Pollen viability	XY335 (2022)		[55]
3	78%	Pollen viability	Zhuyu309	HTS for 5d, starting 15d before silking	[56]
	70%	Pollen viability	Zhuyu309	HTS for 5d, starting 10d before silking	[56]
	78%	Pollen viability	Zhuyu309	HTS for 5d, starting 5d before silking	[56]
	80%	Pollen viability	Zhuyu309	HTS for 5d, starting 5d after silking	[56]
	58%	Pollen viability	Zhuyu309	HTS for 15d, starting 15d before silking	[56]
4	82%	Pollen viability	Zhuyu309		[59]
**Cellular Aspect: Seed setting**					
5	10.74%	Fertilization Rate	Zhengdan 958		[19]
	4.32%	Fertilization Rate	Xianyu335		[19]
6	16.56%	seed-set	BT-1		[27]
	88%	seed-set	N6		[27]
7	80.67%	Seed set	DK752		[30]
	69.69%	Seed set	DK664		[30]
8	70%	Seed set	Xianyu335	BS-2W: HS before silking for 2 weeks	[48]
	15%	Seed set	Xianyu335	2W-BA: HS 2 weeks before and after silking	[48]
	40%	Seed set	Xianyu335	AS-2W: HS after silking for 2 weeks	[48]
9	74%	Seed set	Zhuyu309	HTS for 5d, starting 15d before silking	[56]
	64%	Seed set	Zhuyu309	HTS for 5d, starting 10d before silking	[56]
	70%	Seed set	Zhuyu309	HTS for 5d, starting 5d before silking	[56]
	58%	Seed set	Zhuyu309	HTS for 5d, starting 5d after silking	[56]
	62%	Seed set	Zhuyu309	HTS for 5d, starting 10d after silking	[56]
	70%	Seed set	Zhuyu309	HTS for 5d, starting 15d after silking	[56]
	68%	Seed set	Zhuyu309	HTS for 15d, starting 5d before silking	[56]
	60%	Seed set	Zhuyu309	HTS for 15d, starting 15d after silking	[56]
10	25%	Seed set	B73	HTS for 1h after pollination	[57]
	15%	Seed set	B73	HTS for 4h after pollination	[57]
11	64.5%	Seed set	Zhuyu309		[59]
12	106%	Seed set	DK7210 VT3Pro (Te)	2017	[60]
	99.9%	Seed set	DK7210 VT3Pro (Te)	2018	[60]
	95.6%	Seed set	DK7210 VT3Pro (Te)	2019	[60]
	79.9%	Seed set	DK7710 VT3Pro (St)	2017	[60]
	95.2%	Seed set	DK7710 VT3Pro (St)	2018	[60]
	101%	Seed set	DK7710 VT3Pro (St)	2019	[60]

Table S1. Continued.

13	79.74%	Grain yield	ADA17.4	2020	[50]
	79%	Grain yield	ADA17.4	2021	[50]
	89.7%	Grain yield	SYM-307	2020	[50]
	89.98%	Grain yield	SYM-307	2021	[50]
	83.88%	Grain yield	Check-1	2020	[50]
	86%	Grain yield	Check-1	2021	[50]
14	30.8%	Grain yield	Zhuyu309	HTS for 6h after pollination	[53]
	20.2%	Grain yield	Zhuyu309	HTS for 12h after pollination	[53]
	103%	Grain yield	Zhuyu309	HTS for 6h, starting 6h after pollination	[53]
	88%	Grain yield	Zhuyu309	HTS for 12h, starting 12h after pollination	[53]
	54.%	Grain yield	B73	HTS for 6h after pollination	[53]
	18.1%	Grain yield	B73	HTS for 12h after pollination	[53]
	87.25%	Grain yield	B73	HTS for 6h, starting 6h after pollination	[53]
	81.1%	Grain yield	B73	HTS for 12h, starting 12h after pollination	[53]
**Cellular Aspect: ROS accumulation**					
15	116.6%	ROS content	SYN5		[36]
	109.9%	ROS content	YN7		[36]
**Cellular Aspect: Photosynthetic Efficiency**					
16	90%	Photosynthetic rate	Papirika		[46]
	100%	Photosynthetic rate	Pioneer 221		[46]
17	69.07%	Chlorophyll a	DTPYC9F119		[28]
	34.88%	Chlorophyll b	DTPYC9F119		[28]
	45.95%	Chlorophyll a	K64R		[28]
	23.53%	Chlorophyll b	K64R		[28]
18	31.15%	PSII efficiency	F223		[34]
	50%	PSII efficiency	F250		[34]
19	64.29%	photosynthetic CO_2 (Pn)	F3		[35]
	53%	photosynthetic CO_2 (Pn)	F186		[35]
	64%	photosynthetic CO_2 (Pn)	F212		[35]
	55%	photosynthetic CO_2 (Pn)	F223		[35]
	69%	photosynthetic CO_2 (Pn)	F250		[35]
	54%	photosynthetic CO_2 (Pn)	F469		[35]
20	88.2%	Photosynthetic Efficiency	SYN5		[36]
21	50%	PSII efficiency	Penjalinan		[37]
22	64.7%	Fv/Fm	HS208		[41]
	78.3%	Fv/Fm	SD609		[41]
**Cellular Aspect: Sugar Metabolism**					
23	82%	Starch content	Zhengdan958	Basal kernel	[16]
	76.2%	Starch content	Zhengdan958	Apical kernel	[16]

Table S1. Continued.

24	41.5%	starch content	XY335		[17]
25	97%	Starch content	Suyunuo5	2014	[51]
	96.7%	Starch content	Suyunuo5	2015	[51]
	46.3%	Soluble sugar content	Zhuyu309		[59]
**Cellular Aspect: Antioxidant Enzyme Activity**					
26	168.15%	SOD activity	DTPYC9F119		[28]
	137.95%	SOD activity	K64R		[28]
27	83.4%	SOD activity	SYN5		[36]
	90.1%	SOD activity	YN7		[36]
28	91.1%	POD activity	DH605		[38]
29	79.6%	SOD activity	HS208		[41]
	82.3%	SOD activity	SD609		[41]
30	121%	SOD activity	ZD958		[55]
	104%	SOD activity	Xianyu335		[55]
